# Features of human scabies in resource-limited settings: the Cameroon case

**DOI:** 10.1186/s12895-015-0031-0

**Published:** 2015-07-23

**Authors:** Emmanuel Armand Kouotou, Jobert Richie N Nansseu, Isidore Sieleunou, Defo Defo, Anne-Cécile Zoung-Kanyi Bissek, Elie Claude Ndjitoyap Ndam

**Affiliations:** Biyem-Assi District Hospital, Yaoundé, Cameroon; Department of Medicine and Medical Specialties, Faculty of Medicine and Biomedical Sciences, University of Yaoundé I, Yaoundé, Cameroon; Yaoundé General Hospital, Yaoundé, Cameroon; Sickle Cell Disease Unit, Mother and Child Centre, Chantal Biya Foundation, Yaoundé, Cameroon; Department of Public Health, Faculty of Medicine and Biomedical Sciences, University of Yaoundé I, PO Box 1364, Yaoundé, Cameroon; School of Public Health, University of Montréal, Montréal, Canada; Yaoundé Central Hospital, Yaoundé, Cameroon

**Keywords:** Human scabies, Contagiousness, Post-scabies pruritus, Cameroon

## Abstract

**Background:**

The persistent high prevalence of human scabies, especially in low- and middle-income countries prompted us to research the sociodemographic profile of patients suffering from it, and its spreading factors in Cameroon, a resource-poor setting.

**Methods:**

We conducted a cross-sectional survey from October 2011 to September 2012 in three hospitals located in Yaoundé, Cameroon, and enrolled patients diagnosed with human scabies during dermatologists’ consultations who volunteered to take part in the study.

**Results:**

We included 255 patients of whom 158 (62 %) were male. Age ranged from 0 to 80 years old with a median of 18 (Inter quartile range: 3–29) years. One to eight persons of our patients’ entourage exhibited pruritus (mean = 2.1 ± 1.8). The number of persons per bed/room varied from 1 to 5 (mean = 2.1 ± 0.8). The first dermatologist’s consultation occurred 4 to 720 days after the onset of symptoms (mean = 77.1 ± 63.7). The post-scabies pruritus (10.2 % of cases) was unrelated to the complications observed before correct treatment (all p values > 0.05), mainly impetiginization (7.1 %) and eczematization (5.9 %).

**Conclusion:**

Human scabies remains preponderant in our milieu. Populations should be educated on preventive measures in order to avoid this disease, and clinicians’ knowledges must be strengthened for its proper diagnosis and management.

## Background

Human scabies, an ectoparasitosis transmitted to humans through direct or indirect skin contact, is caused by *Sarcoptes scabiei hominis*, a mite infecting only human beings [1]. This skin infection has been described to occur as cyclic epidemics [2]. It has also been shown to be associated with poverty, overpopulation, poor personal hygiene, and war-centric pandemics [1–3]. In fact, the disease is particularly rampant in overcrowded places without adequate sanitation such as school milieu, nursing homes and prisons [2, 4]. The diagnosis of scabies is based on identification of the mite using dermoscopy and/or skin scrapings/microscopy. But in resource-constrained areas where these technologies may scarcely be available, the diagnosis of scabies will be essentially based on anamnesis and clinical findings, hence the need for an experienced clinician who will make the right diagnosis [3, 4]. The management of this pathology integrates treatment of the patient as well as that of his entourage, along with disinfection of clothes and bedding [4].

Evidence from the literature shows that the prevalence of scabies in African countries is persistently high, being as such noticeable among individuals, and in some specific groups and collectivities [3–6]. As a matter of fact, nearly 300 million cases of this skin infection are reported in the world annually [7]. As such, scabies is one of the pathologies mostly encountered in developing and resource-limited countries [8, 9].

Paradoxically, there is dearth of research targeting the features of human scabies in resource-poor countries. The present study was thus undertaken, the purpose of which was to draw up the socio-demographic profile of patients infected with scabies, and to determine its related spreading factors in Cameroon, a low-income country.

## Methods

### Study design and setting

We conducted a cross-sectional survey from October 2011 to September 2012 in 3 hospitals, all of which are located in Yaoundé the political capital of Cameroon, namely: the Yaoundé Central Hospital, the Biyem-Assi District Hospital and the Elig-Essono Medical Centre. Each of these three health facilities are provided with competent and experienced dermatologists who are lecturers in the main Faculty of Medicine of the country.

### Study participants and data collection

Participants were patients, irrespective of their age and sex, who came or were brought on out-patient consultations for dermatologic problems. During the survey period, we consecutively included patients who described night-prevailing pruritus and/or any notion of contamination, and whose physical examination, performed by the dermatologist, revealed characteristic lesions of human scabies.

Data collection used a structured questionnaire recording socio-demographic background (age, sex, profession, educational level, underlying conditions), risk or spreading factors (number of persons living in the room or sleeping on the same bed), duration between onset of symptoms and first consultation, and clinical relevant signs and symptoms.

After the diagnosis was made at this initial consultation, patients were placed on specific medication, namely the benzoate of benzyle to be applied on the whole body two times at a 24 h-interval, after a lukewarm bath and on a humid skin. This operation was to be repeated one week later. Patients and their entourage were treated simultaneously. In cases of impetiginization, an antiseptic and a macrolide were prescribed for seven days before the application of the scabicide, and in cases of eczematization, a topic corticoid was to be applied 24 h after the anti-scabious treatment for 4 to 5 days. Additionally, clothes and bedding were decontaminated by washing them with warm water followed by spraying of an anti-scabious containing pyréthrinoïde. No quarantine measures were applied. Patients were subsequently given a weekly appointment to assess evolution of lesions and response to treatment. Unhappily, dermoscopy and/or skin scrapings/microscopy were not available to confirm the presence of mite. Therefore, we were comforted in our diagnosis in case the response to treatment was good with complete regression of lesions. The post-scabies treatment pruritus was considered as persistent after three completed weeks following an adequate anti-scabious treatment.

### Statistical methods

Data were coded and entered using Microsoft Excel 2010 from Windows, and were further analyzed with Epi info version 3.5.3 (Centre for Disease Control, Atlanta, USA). Results are presented as mean ± standard deviation (SD) or median (inter-quartile range IQR) for quantitative variables, and as count (frequency) for qualitative ones. The Chi-2 test served for qualitative variable comparisons and the Student *t* test (or equivalents) for quantitative ones. Odds ratios (OR) with 95 % confidence intervals (CI) were used to investigate the influence of eczematisation and impetiginization on the persistence of pruritus after treatment. A p value < 0.05 determined statistically significant results.

### Ethical considerations

Approvals were obtained from administrative authorities of the different study sites, and an ethical clearance was delivered by the ethical board of the Faculty of Medicine and Biomedical Sciences of the University of Yaoundé I, Cameroon. Patients or their guardians were informed of the various aspects of the study, and we anonymously enrolled only those who volunteered to take part in it after they have signed an informed consent form.

## Results

### Background characteristics

On the whole, we recruited 255 patients diagnosed with scabies, among whom 158 (62 %) were males, hence a sex ratio of 1.63/1. Participants’ ages ranged from 0 to 80 years old, with a median of 18 (IQR 3-29). Table 1 displays our participants’ age groups, educational level, profession and health underlying conditions. Patients aged 0-5 years old were the most encountered (30.6 %) followed by those aged 25–35 years (23.1 %, see Table 1). Ninety patients (35.3 %) attended the secondary school, and 46 (18 %) went to the university or college. Forty three point one percent of our respondents were students, and 17 (6.7 %) were civil servants. One patient (0.4 %) presenting with scabies (specifically Norwegian scabies) was mentally retarded, and 4 patients (1.6 %) were known HIV positive subjects (see Table 1).

**Table 1:** Characteristics of the study population with regard to their age, educational level, profession and underlying condition

		Number	Percentage (%)
Age (years)	<1	30	11.8
	1–5	48	18.8
	6–11	27	10.6
	12–18	25	9.8
	19–24	28	11
	25–35	59	23.1
	36–45	16	6.3
	> 45	22	8.6
Educational level	Never went to school	5	2.0
	Nursery	17	6.7
	Primary	45	17.6
	Secondary	90	35.3
	University/College	46	18.0
	Missing data	52	20.4
Profession	Civil servant	17	6.7
	Driver	6	2.4
	Trader	13	5.1
	Couturier/Hairdresser	11	4.3
	Student	110	43.1
	Teacher	4	1.6
	Manoeuvre	12	4.7
	Housewife	11	4.3
	Other	8	3.1
	Unemployed	11	4.3
	Missing data	52	20.4
Underlying condition	Retarded person		
	Yes	1	0.4
	No	254	99.6
	HIV infection		
	Yes	4	1.6
	No	103	40.3
	Unknown	144	56.5
	Missing data	4	1.6

### Spreading factors

When analyzing the spreading factors we have searched for (see Table 2), we found no age difference between males and females. Two hundred and forty two subjects (94.9 %) were currently living with their family members, and almost all our participants were suspected to have been contaminated by their close entourage. The number of persons per room or bed varied from 1 to 5 with a mean of 2.1 ± 0.8, and there was no related difference between men and women (*p* = 0.51). Furthermore, the number of persons exhibiting pruritus in the entourage ranged between 1 and 8 with a mean equal to 2.1 ± 1.8, and was significantly higher among males than females (2.5 ± 2.0 vs 1.9 ± 1.5 respectively, *p* = 0.02; see Table 2). Duration between onset of symptoms and first consultation varied from 4 to 720 days, with a mean of 77.1 ± 63.7. Before this consultation, 195 patients (74.9 %) had already tried a previous treatment without any success (mainly antibiotics, antifungals, antihistaminics or plant-based medicines), this being either prescribed by general practitioners or specialists other than dermatologists, or auto-medications.

**Table 2:** Spreading factors compared between males and females

Variable	Sex (mean ± SD)		Total	*p*
	Female	Male		
Age	2.1 ± 0.7	19.6 ± 18.3	2.1 ± 0.7	0.76
Number of persons per bed/room	2.1 ± 0.7	1.9 ± 0.8	2.1 ± 0.8	0.51
Number of persons with pruritus in the entourage	2.5 ± 2.0	1.9 ± 1.5	2.1 ± 1.8	0.02*
Duration between onset of symptoms and first consultation (days)	79.0 ± 64.9	76.0 ± 63.2	77.1 ± 63.7	0.72

*p value < 0.05

### Results of physical examination

The main lesions we observed were crusts (82.4 %), papules (69.8 %), and papulo-vesicles (68.6 %; see Table 3). These lesions were predominantly located at interdigital spaces (80 %), under-buttock creases (71.8 %), wrists (70.2 %), and inter-buttock creases (56.5 %; see Table 3). Only one patient (0.4 %) presented with crusted/Norwegian scabies. Two skin related complications were recorded before treatment: impetiginization (7.1 %) and eczematization (5.9 %). Four patients (26.7 %) presenting with eczematization had a history of atopy. After being adequately treated (i.e. anti-scabies + disinfection of clothes and bedding + treatment of the whole entourage) with complete regression of lesions, twenty six (10.2 %) patients presented with persistence of pruritus. While investigating the influence of impetiginization and eczematization on the occurrence of post-scabies pruritus, we found that patients with eczematization before an adequate treatment would have had an odds of 2.1 to develop a post-scabies pruritus than their counterparts, and an odds of 1.1 in case of impetiginization. But these odds are merely descriptive, as they were statistically non-significant (*p* = 0.19 and 0.57 respectively; see Table 4).

**Table 3:** Type and location of lesions

	Number (*N* = 255)	Percentage (%)
Type of lesions		
Vesicles	137	53.7
Papulo-vesicles	175	68.6
Papules	178	69.8
Nodules	65	25.5
Pustules	40	15.7
Crusty lesions	210	82.4
Scabious furrow	55	21.6
Scratch marks	135	52.9
Location of lesions		
Face	25	9.8
Anterior trunk	123	48.2
Posterior trunk	109	42.7
Axilla	136	53.3
Areola	60	25.5
Umbilical	99	38.8
Wrist	179	70.2
Under-buttock creases	183	71.8
Inter-buttock creases	144	56.5
Palms	45	17.6
Interdigital spaces	204	80.0
Glans	118	46.3
Sole	40	15.7
Diffuse	51	20.0

**Table 4:** Factors influencing the persistence of pruritus after treatment

		Persistence of pruritus		OR	95 % CI		*p* value
		Yes	No				
Eczematization							
	Yes	3	12	2.1	0.7	6.3	0.19
	No	22	210	1	–	–	
Impetinization							
	Yes	2	16	1.1	0.3	4.3	0.57
	No	23	206	1	–	–	

*OR* Odds ratio, *CI* Confidence interval

## Discussion

This hospital-based cross-sectional study among an out-patient population revealed that human scabies may be common in Yaoundé, Cameroon. The number of persons per room/bed as well as the number of persons in the entourage may play a role in the spreading of the disease, though the design of the study precluded us from meticulously investigating such interactions. Further, the dominating lesions were crusts, papules and papulo-vesicles, and the prevailing sites of lesions were buttocks and wrists. We do suggest therefore that populations, especially those living in resource-limited areas, should be educated on preventive measures such as adopting rigorous personal hygiene, avoiding overcrowding and overpopulation in rooms/houses whenever possible, and promoting or reinforcing hand hygiene. Additionally, there is need to emphasize on scabies training for medical students and introduce regular updates on scabies diagnosis and treatment for health care workers. Eventually, Governments should work towards poverty reduction to limit overcrowding.

We found a male (62 %) and younger age (median 18 years) predominance, these being consistent with Do Ango-Padonou et al’s findings in Benin [10]. More than half of our respondents (59.6 %) had attended the nursery, primary or secondary school, but only 46 (18 %) patients had gone to the university. This finding may be due to the fact that younger children have closer physical contact with more individuals; as such they may be at increased risk of infestation. But further studies are warranted to thoroughly investigate such a concern. On another hand, we recorded 110 students (43.1 %), comparable to the 50.8 % proportion recorded in Bangui and 41.7 % in Dakar [11, 12]. This predominance of students could perhaps be explained by the overcrowding and overpopulation that characterize schools of resource-limited contexts, as there is body of evidence claiming that these are, among others, prevailing risk factors of human scabies [2–4, 6].

Consistent with the literature, results from our study are perhaps suggestive that overcrowding may be a contributive factor leading to the occurrence of scabies [3, 4, 12, 13]. Indeed, we found that 242 (94.9 %) of our patients were currently living with other members of their families, with more than one person sharing the same room/bed, and more than one person exhibiting pruritus in the entourage. It is true however that we must have undertaken a case-control study to underpin such an observation with robust scientific evidence.

We observed a delay of 4 to 720 days from onset of symptoms to the first dermatologist consultation. This may be attributable to the low socio-economic status of our patients as we are in a resource-poor context, and less than 10 % of our participants had a constant salary. Further, shame and/or taboo may delay the consultation, given scabies is perceived to be associated with poor hygiene and sanitation [2–4]. Lastly, non-dermatologist physicians may contribute to this delay if they are not well trained to properly diagnose and manage or refer cases of scabies. Such a long delay we have witnessed from onset of symptoms to diagnosis and adequate treatment is an important contributive factor in the spreading of the disease, the patient remaining contagious during all this period. It may consequently explain the persistently high prevalence of the disease latterly described [6, 13, 14].This constant high prevalence of scabies among resource-limited settings, along with its legendary contagiousness and related complications, makes perhaps this pathology a real public health hazard which may deserve full attention from local health authorities [15].

Evidence from the literature shows that the diagnostic of human scabies may be essentially clinical with a very good sensitivity (96.2 %) and specificity (98.0 %) [15]. The disease may evolve to a chronic stage, this fuelled by a long delay between onset of symptoms and adequate management, presenting thereby with some complications. For instance, we found that 7.1 % and 5.9 % of our patients respectively exhibited impetiginization and eczematization. But Kobangué et al. [11] observed a 2 to 3 times higher prevalence of these complications than ours: 17.2 % and 19.9 % respectively for the former and the latter. Besides, 10.2 % of our respondents exhibited a persistence of pruritus after they have been adequately treated and followed-up. This finding is in line with previous observations and has been attributed to the use of irritating drugs to treat the disease, a very bad observance of the treatment, an acarophobia, or an early and premature re-infestation [16].

While examining the influence of impetiginization and eczematization on the occurrence of post-scabies treatment pruritus, we found that patients with eczematization before any adequate treatment would have been 2.1 times more likely than their counterparts to develop a post-scabies pruritus, and, 1.1 times in case of impetiginization. Regrettably, these findings were not statistically significant (*p* = 0.19 and 0.57 respectively), may be due to the cross-sectional design of the study and the small sample size. Over time, the pruritus, which is sometimes very disabling, alongside the chronic lesions and complications of scabies, may disastrously impact the health condition of patients, dreadfully altering the quality of life of children and adults as well [8, 9].

Unfortunately, the design of this study precluded us from extensively investigating risk factors of scabies in our milieu. In fact, there was no control group (e.g. patients presenting with skin conditions, but without scabies) in order to compare the two groups and sort out some risk factors. Additionally, we are unable to generalize our findings to the entire Cameroonian population as the study was a hospital-based one, restricted in only three hospitals of Yaoundé, and only to dermatologist consultations too. Moreover, the absence of exact and reliable data on the total number of hospital visits or total number of visits due to skin problems hampered an estimation of the caseload of scabies. Another limitation of this study lies in the small sample size which could perhaps explain the absence of associations between variables. Eventually, dermoscopy and/or skin scrapings/microscopy were not available to confirm the presence of mite, and we did not search for proteinuria in patients presenting with impetiginization. Nonetheless, the two dermatologists in charge of the clinical assessment of the disease were very well-trained and experienced clinicians, and the clinical assessment of scabies has been shown with good sensitivity and specificity [16]. Further well-designed studies with large number of patients need to be conducted in order to better assess the burden, risk factors and clinical profile of human scabies in our settings.

## Conclusion

To date, human scabies remains a common dermatologic pathology in Yaoundé, Cameroon. There are contributive factors such as an increased number of persons per room/bed or in the close entourage, and a long delay between onset of symptoms and proper diagnosis and management. Populations must be educated and sensitized on its related preventive measures such as adoption of rigorous personal hygiene, avoidance of overcrowding and overpopulation in rooms/houses whenever possible, and promotion or reinforcement of hand hygiene. Additionally, there is need to increase emphasis on scabies training for medical students and introduce regular updates on scabies diagnosis and treatment for health care workers. Eventually, Governments should work towards poverty reduction to limit overcrowding.
